# Personalized Approach to Olfactory Neuroblastoma Care

**DOI:** 10.3390/jpm14040423

**Published:** 2024-04-16

**Authors:** David K. Lerner, James N. Palmer

**Affiliations:** Department of Otolaryngology-Head and Neck Surgery, University of Pennsylvania, Philadelphia, PA 19104, USA

**Keywords:** olfactory neuroblastoma, endoscopic sinus surgery, sinonasal tumors, endoscopic skull base surgery

## Abstract

Olfactory neuroblastoma (ONB) is an uncommon neuroendocrine malignancy arising from the olfactory neuroepithelium. ONB frequently presents with nonspecific sinonasal complaints, including nasal obstruction and epistaxis, and diagnosis can be obtained through a combination of physical examination, nasal endoscopy, and computed tomography and magnetic resonance imaging. Endoscopic resection with negative margins, with or without craniotomy, as necessary, is the standard of care for definitive treatment of ONB. Regional metastasis to the neck is often detected at presentation or may occur in a delayed fashion and should be addressed through elective neck dissection or radiation. Adjuvant radiotherapy should be considered, particularly in the case of high grade or tumor stage, as well as positive surgical margins. Systemic therapy is an area of active investigation in both the neoadjuvant and adjuvant setting, with many advocating in favor of induction chemotherapy for significant orbital or intracranial involvement prior to surgical resection. Various targeted immunotherapies are currently being studied for the treatment of recurrent or metastatic ONB. Prolonged locoregional and distant surveillance are indicated following definitive treatment, given the tendency for delayed recurrence and metastasis.

## 1. Introduction

Olfactory neuroblastoma (ONB) is a rare neuroendocrine malignancy arising from the olfactory neuroepithelium that is estimated to comprise approximately 5% of all sinonasal malignancies [1,2,3,4]. ONB has traditionally been considered to present in a bimodal age distribution, but recently larger studies have demonstrated a wide range of age presentations, with a peak between the fourth and sixth decades [5]. There are no known risk factors for the development of ONB [2].

Clinical signs and symptoms vary depending on the extent and area involved by each individual tumor. The olfactory neuroepithelium is located at the superomedial aspect of the nasal cavity and is in close proximity to the skull base and intracranial compartment superiorly, as well as the orbits laterally to each side. In general, the most commonly reported presenting symptoms for ONB include nasal airway obstruction and epistaxis. However, tumors that extend to involve the orbit or orbital apex may present with diplopia, proptosis, or visual changes, while tumors that extend intracranially can present with headache, nausea and vomiting, as well as a range of cranial neuropathies [1,3,6]. The diagnosis of ONB can be quite challenging for providers, as small tumors that only involved the nasal cavity tend to present with nonspecific symptoms, and therefore, diagnosis and appropriate specialist referral is often delayed [7].

**Figure 1:**
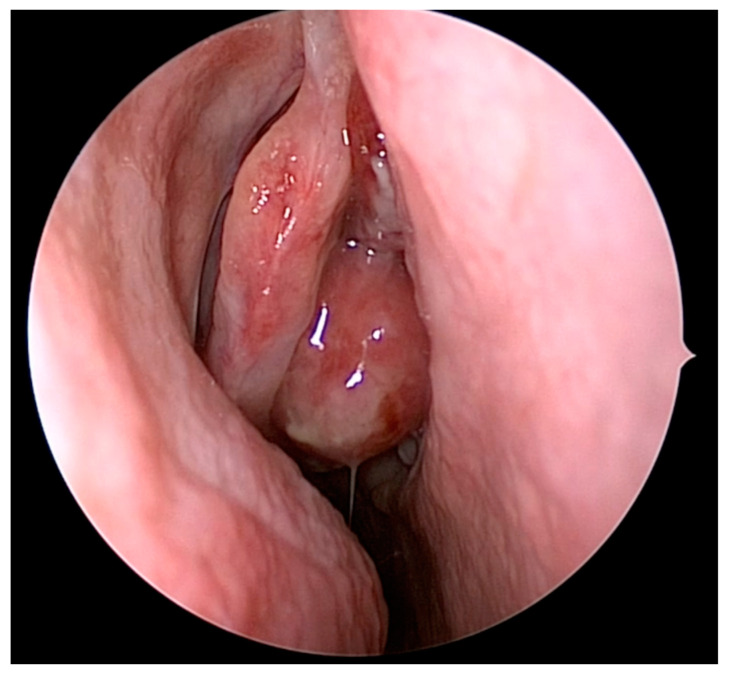
Olfactory neuroblastoma visualized during nasal endoscopy. The mass is located medial to the patient’s right middle turbinate, extending from the olfactory cleft.

**Figure 2:**
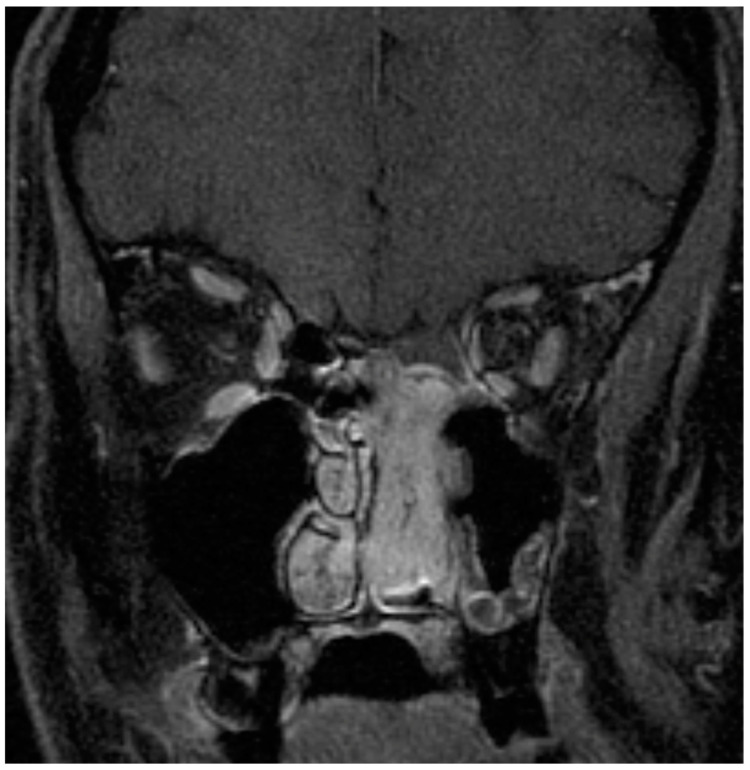
Magnetic resonance imaging for the diagnosis of olfactory neuroblastoma. A Kadish B tumor is seen here involving the nasal cavity and paranasal sinuses, not the orbits or intracranial cavity.

As with the diagnosis of any sinonasal mass, physical examination and nasal endoscopy are essential in the diagnosis and evaluation of ONB. A sample endoscopic view of an ONB arising from the olfactory cleft is depicted in Figure 1. Endoscopically, ONB are typically characterized by a fleshy or friable appearance of a mass that appears to arise from the olfactory cleft and likely in continuity with the skull base. A combination of computed tomography (CT) and magnetic resonance imaging (MRI, Figure 2) are routinely obtained in the diagnosis of ONB. CT is useful to evaluate the extent of the disease, as well as associated osseous destruction within the nasal cavity and of the orbit and skull base. MRI is useful to evaluate the extent of the disease, as well, but also provides better information than CT regarding orbital and intracranial involvement and dural involvement and perineural spread. On MRI, ONB is typically hypointense on T1-weighted imaging sequences and intermediate to hyperintense of T2-weighted imaging sequences. The T2 sequence should be examined to best distinguish tumors from trapped secretions, which are characteristically quite hyperintense [8].

Critical features to examine during a review of preoperative sinonasal imaging regarding the extent of disease, including the presence of intracranial or orbital extension, as well as whether there is unilateral or bilateral disease within the nasal cavity. Fat-saturated MRI sequences are particularly useful for delineating the extent of orbital involvement, specifically providing detail as to whether there is a pushing or infiltrating interface between the tumor and orbital contents. Even if there is not gross intracranial tumor extension on MRI, it is essential to evaluate for dural infiltration on preoperative imaging, as this finding has significant negative prognostic implications [8,9,10]. Special attention should be paid to the neck on imaging to evaluate for the presence of cervical metastasis, which has been noted in around 10% of patients on diagnosis, most commonly presenting in level II of the neck [11]. In terms of the evaluation of distant disease, positron emission tomography–computed tomography (PET-CT) may also be obtained after tissue diagnosis to evaluate for both regional and distant metastasis [1,8]. The traditional radiolabeled 18-fluorodeoxyglucose (FDG) PET-CT has been shown to be useful for assessing distant disease in the initial workup for ONB, as well as for detecting recurrences. However, the utility of FDG PET-CT is limited by the relatively low metabolic activity of ONB compared to other sinonasal cancers. Given that ONB expresses high levels of somatostatin receptors, targeted radiotracers, such as gallium-68 and lutetium-177 dotatate, have been proposed as more specific markers. Studies with limited sample sizes have shown promising utility in the detection of small residual or recurrent locoregional and distant disease, but further studies with larger sample sizes will be necessary to further elucidate the role of these alternate imaging modalities [12,13,14,15,16].

The mainstay of treatment for ONB is surgical resection with or without adjuvant radiotherapy and chemotherapy. During workup for ONB, special attention should be paid to the local tumor extent, specifically the degree of involvement of the intracranial and orbital compartments, as well as the presence of regional or distant disease as detected on imaging. Ultimately, decision-making regarding surgical approach and adjuvant treatment depends on a range of patient- and tumor-specific factors and is tailored to each individual patient [1,8,17].

## 2. Patient- and Tumor-Specific Factors

Among all patients, 5- and 10-year survival rates for ONB were 62.1% and 45.6% in the Surveillance, Epidemiology, and End Results (SEER) database [18]. However, each provider must consider a range of patient- and tumor-specific factors that may influence outcomes in the care of patients with ENB.

A Carey et al. review of the National Cancer Database examined patient-specific factors that affect ONB patients and found that patients older than 65 years of age have poorer all-cause mortality than patients under 54 years old, and patients with a higher comorbidity burden as reflected by high Charlson–Deyo scores have a higher all-cause mortality [19]. Female gender has also been associated with improved outcomes relative to male gender, although this is considered to be attributable to poorer tumor stage and grade at diagnosis [19,20].

The Hyams grading system is a widely used system of tumor stratification and prognostication based on histopathological analysis [2,21]. The Hyams system incorporates five different pathological features into a grade I–IV scale, which is typically reported as “low-grade” (grades I–II) or “high-grade” (grades III–IV) [1]. Characteristic histopathologic features of ONB include considerable microvascularity, as well as Homer–Wright pseudorosettes in low-grade tumors and Flexner–Wintersteiner rosettes in high-grade tumors. Low- and high-grade tumors also vary in the degree of necrosis, mitoses, and nuclear pleomorphism [16].

The utility of the Hyams system as a means of prognostication has been widely confirmed across a range of retrospective studies and meta-analyses. Overall, high-grade tumors have been variably linked with more aggressive locoregional disease, as well as poorer disease-free survival and overall survival relative to low-grade tumors [2,17,21,22,23,24,25].

A variety of staging systems have historically been used for ONB, starting with the Kadish staging system in 1976 [26] (Table 1). The Kadish system was expanded upon by Morita et al. to create a “modified Kadish” staging system that incorporated cervical and distant metastasis as a separate stage (Table 1) [27]. Separately, in 1992, Dulguerov et al. proposed a new T-staging system with a focus on radiological findings [28] (Table 2). In general, a more advanced clinical stage is associated with worse survival outcomes [9,19,25,29,30,31]. However, studies comparing the modified Kadish and Dulguerov staging systems have drawn mixed results as to the superior approach for prognostication [9,10].

More recently, a Joshi et al. analysis of the SEER database identified dural infiltration or invasion as notable independent prognostic features [2,9]. As a result of these findings, there has been an effort to update the Kadish staging system into a Kadish-INSICA staging system. In this outcome-based approach, the Kadish stages A and B are combined into a new Kadish-INSICA stage A, and the traditional Kadish stage C is subdivided into tumors without (Kadish-INSICA B) and with dural infiltration (Kadish-INSICA C) on preoperative imaging [10]. Stage D tumors remain those with nodal or distant metastases in this system. Notably, clinical stages within the Kadish-INSICA system appear to align well with overall survival [10]. Overall, each staging system represents an effort to standardize the extent of tumor involvement and provide useful prognostic information for each individual patient, despite the lack of a single universally accepted system.

Both Hyams grade, as well as clinical stage, have implications for the necessity and utility of adjuvant treatment, as discussed below.

## 3. Primary Tumor Treatment

The standard of care treatment for ONB is surgical resection with negative margins [1]. Definitive surgical resection has been traditionally accomplished via an open craniofacial resection, but over recent decades, an endoscopic resection has become the preferred approach for amenable tumors [32,33]. When planning surgical resection, the approach should be tailored to the specific patient and tumor; options include the traditional open craniofacial approach, the endoscopic-only approach, and the endoscopic-assisted approach, in which the standard endoscopic approach is paired with a bifrontal craniotomy to address significant intracranial tumor extension. In general, the necessity of an open approach should be considered for patients with tumors that extend laterally to the midpupillary line, involve facial soft tissue, or extensively involve the intraorbital or intracranial compartments. For tumors that involve the skull base, treatment plans are typically developed and implemented in tandem between the otolaryngologist and neurosurgeon.

A 2021 Barinsky et al. analysis of the National Cancer Database reported that, from 2010 to 2015, there was a nearly even split among patients undergoing open and endoscopic surgical approaches for ONB resection. They reported that patients with Kadish stage A and B tumors were significantly more likely to undergo endoscopic resection rather than an open approach and that these patients had, on average, a shorter postoperative hospital stay. Notably, patients undergoing an endoscopic approach to tumor resection demonstrated a significantly improved 5-year overall survival rate (81.9% vs. 75.6%). These findings reflect that patients without significant local invasion or distant spread are excellent candidates for an endoscopic approach [34]. Similarly, Fu et al. reported higher overall survival, as well as disease-specific survival rates, for all patients, as well as Kadish stage C/D and high-grade Hyams subgroups, in a systematic review and meta-analysis [32]. Within this study, the authors reported lower rates of intracranial complications and total complications within the endoscopic resection cohort, in addition to postoperative cerebrospinal fluid leak rates that were not significantly different [32]. Lastly, a 2017 Harvey et al. staged-matched comparison between open transcranial resection compared to endoscopic resection of ONB reported favorable survival outcomes for patients undergoing endoscopic resection [33]. Overall, the endoscopic approach is the preferred approach for cases in which margin-negative resection is achievable.

The orbit must be assessed for tumor involvement when devising a treatment plan for each individual patient. ONB is known for its tendency to invade local structures, and studies have reported rates of orbital invasion ranging from 10 to 38% [35,36,37]. Orbital invasion itself has been demonstrated across a number of studies to be a negative prognostic indicator that is associated with worse overall survival, as well as disease-free survival [35,37]. Traditionally orbital involvement has prompted more aggressive up-front surgery via open craniofacial resection, but there has been a recent movement toward orbital preservation when feasible [38,39]. In cases of significant local orbital involvement, many have advocated for a limited resection, including resecting periorbita or the lacrimal sac without addressing the orbit proper, followed by adjuvant therapy [40,41]. Others have advocated for induction chemotherapy for tumors with significant local invasion into the orbit and have reported cases of successful and durable orbital preservation with induction chemotherapy, particularly among patients with Hyams grade III or IV tumors [11,41,42]. The 2023 International Consensus Statement of Allergy and Rhinology: Sinonasal Tumors has deemed induction chemotherapy for advanced ONB with significant orbital invasion to be a treatment option, particularly for high-grade tumors [2]. Further studies will be needed to further evaluate the safety and efficacy of this approach.

Complications from the resection of ONB vary between open and endoscopic approaches, as well as based on the areas involved in tumor resection. Serious complications include orbital, vascular, and intracranial injuries among others [18]. Orbital complications range from injury to the nasolacrimal duct, extraocular muscles, and optic nerve, as well as bleeding within the orbital compartment that may result and increased intraocular pressure and, ultimately, visual loss. Vascular injuries vary in severity and include epistaxis, intracranial bleeding, and stroke. Although quite rare, intracranial hemorrhage may arise from injury to branches of the anterior cerebral artery. Intracranial injuries include postoperative cerebrospinal fluid leak and meningitis. Historically, postoperative cerebrospinal fluid leak rates have been high among patients undergoing endoscopic approaches to ONB resection. However, over recent years, postoperative rates of CSF leak have significantly improved with refined surgical techniques and the widespread implementation of pedicle nasoseptal flaps for reconstruction [1,2,18].

## 4. Olfactory Preservation and Surgical Resection of ONB

The primary goal of surgical resection for ONB is a negative margin resection. However, recently, there have been efforts to preserve olfaction during surgical resection for tumors that allow for the preservation of the contralateral olfactory apparatus while achieving a negative margin resection [2,43,44]. A central portion of both traditional open transcranial and endoscopic ONB resections has been the bilateral removal of the cribriform plate, thereby causing permanent and complete olfactory loss. Notably, several single- and multi-institutional studies have been conducted reporting favorable olfactory and oncologic outcomes after unilateral endoscopic ONB resection. For example, Nakagawa et al. first reported a 12-patient series across Dulguerov T1, T2, and T3 stages who underwent negative margin endoscopic resection [45]. All patients had intact smell following surgery, and just one of the nine patients who underwent adjuvant radiotherapy lost their sense of smell following adjuvant treatment. Moreover, no recurrences were reported with a median follow-up time of nearly 44 months [45]. Additionally, Tajudeen et al. conducted a multi-institutional retrospective review incorporating 14 patients undergoing endoscopic unilateral ONB resection across all Kadish stages [43]. Using the University of Pennsylvania Smell Identification Test as a quantitative measure of olfactory dysfunction, Tajudeen et al. demonstrated a 43% rate of residual smell in this cohort in which all patients received adjuvant radiotherapy and four patients additionally received adjuvant chemotherapy. No recurrences were reported with an average follow-up time of over 4 years [43].

Two separate studies have sought to investigate the oncological implications of performing a unilateral ONB resection. Van Gompel et al. surveyed five different skull base surgeons as to whether they were able to accurately predict involvement of the contralateral olfactory apparatus based on preoperative imaging with comparison to histopathological diagnosis, finding that surgeons were able to correctly predict olfactory bulb or tract involvement in 96% of cases [44]. On the other hand, Gomez Galarce et al. performed a cadaveric analysis of 17 specimens to characterize the connectivity of the olfactory system, finding that nearly nine-tenths of specimens had olfactory fibers that crossed from one side to the other, 20% of which crossed along the nasal septum. The authors concluded that there may be some potential oncologic vulnerability in a unilateral resection aiming to spare contralateral olfaction among patients with unilateral tumors and septal involvement [46].

Above all, the most important objective in ONB surgery is achieving a negative margin resection when feasible. The efficacy and oncologic feasibility of unilateral resection to preserve olfaction remains an area of ongoing investigation.

## 5. Treatment of the Neck

Regional metastasis to the neck has been historically associated with considerably worse outcomes [1,2,39,47]. A Nalavenkata et al. multicenter retrospective study identified a 7.1% incidence of neck disease at presentation [47]. An advanced Hyams grade was associated with neck disease at diagnosis, while positive surgical margins were associated with a heightened risk of neck disease presenting in a delayed manner [47]. Similarly, Kuan et al. identified a cervical nodal metastasis rate of 8.7% on presentation, most commonly to cervical level II, and found associations between regional metastasis and both male sex and higher tumor grade [48]. However, in contrast to previous studies, Kuan et al. did not find the presence of neck disease alone to be a significant predictor of overall or disease-free survival on multivariate analysis, suggesting that the poor outcomes associated with the presence of neck disease likely arise secondary to other tumor and patient factors [2,48].

The neck should be evaluated for cervical metastasis during the initial workup. Patients with neck disease are typically recommended for neck dissection, in addition to adjuvant radiation to the neck. Even for those without evidence of neck disease on physical examination or imaging, elective neck radiation or neck dissection should still be considered for high-grade Hyams or Kadish C/D tumors due to the elevated risk of delayed cervical metastasis in this population [1,2]. A McMillan et al. study reported a 22.4% rate of delayed neck recurrence at a median of 57 months, including as delayed as 20 years after the initial presentation [37]. Even for patients without neck disease on presentation, patients should undergo long-term active surveillance of the neck, given the risk of delayed regional metastasis [37,39].

## 6. Posttreatment Surveillance and Salvage Therapy for Recurrence

**Figure 3:**
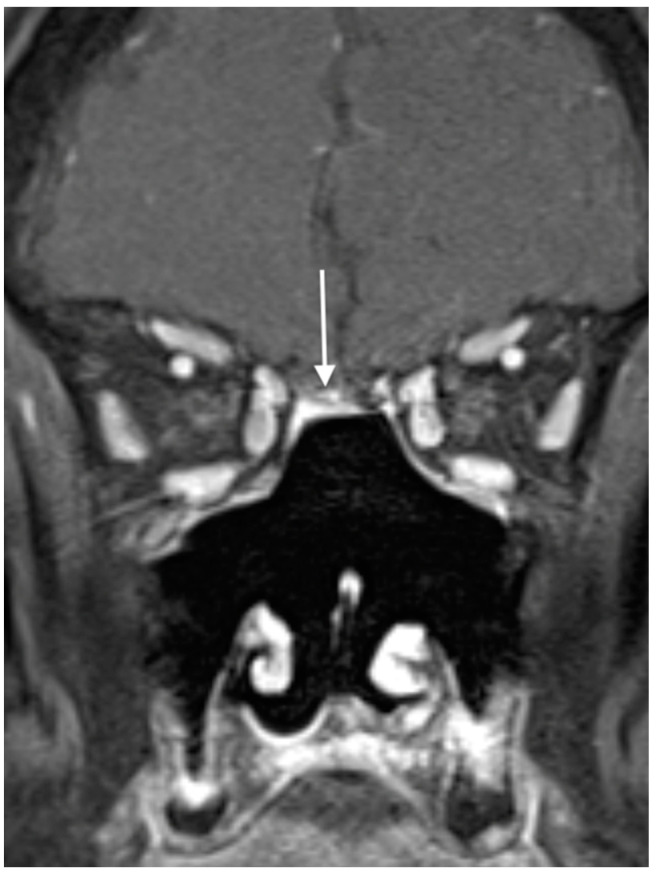
Magnetic resonance imaging for the surveillance of olfactory neuroblastoma. A postresection cavity is seen here without evidence of recurrent disease. The white arrow indicates persistent enhancement of a pedicled nasoseptal flap used in reconstruction.

While there are no official guidelines for posttreatment surveillance, extended posttreatment surveillance is necessary following the definitive treatment of ONB, given its tendency toward delayed locoregional recurrence, as well as late regional and distant metastasis [1,38]. In a Rimmer et al. review of 95 ONB patients, local recurrence was 25.3% overall and occurred most commonly within the first year after treatment. That being said, another 8.3% of local recurrences were detected over a decade following treatment [38]. In terms of regional recurrence, a Wolfe et al. study identified an 18% incidence of delayed neck disease diagnosed at a median of 59 months following treatment [24]. Lastly, the rates of delayed distant metastases have been reported, ranging from 8 to 25% [38]. As a result, surveillance through a combination of physical examination, nasal endoscopy, and imaging evaluating for local, regional, and distant disease are warranted for at least 10 years following treatment [1] (Figure 3).

A Ni et al. retrospective study found the five-year overall survival rate and progression-free survival for patients with recurrent ONB to be 63% and 56%, respectively [49]. Patients with recurrent tumors may present with local, regional, or distant diseases. Each specific patient and tumor should be evaluated for the appropriateness of salvage therapy. For example, a localized tumor recurrence within the sinonasal cavity may be excised endoscopically with limited morbidity. Similarly, a cervical nodal recurrence may be treated with a neck dissection. However, other recurrent diseases that may not be easily and definitively excised should be considered for radiation therapy or systemic therapy in combination with medical and radiation oncology colleagues. Patients with a single recurrence remain at a high likelihood of a second recurrence and should continue to be monitored with a combination of local, regional, and distant surveillance through a combination of clinical evaluation and imaging [1,2,49].

## 7. Adjuvant Treatment

Radiation therapy has been studied for the treatment of ONB as a definitive treatment, as well as in both the neoadjuvant and adjuvant settings. Radiation therapy as a primary treatment is likely most effective for Kadish A tumors in particular [50,51]. However, radiotherapy is most commonly utilized for ONB patients in the adjuvant setting following surgical resection [2]. Radiation has been widely studied for ONB across all tumor stages, but recent works have suggested that radiation may confer a survival benefit only to those with Kadish C or D tumors [30,52]. Overall, radiation is most commonly used in the adjuvant setting following surgical resection, particularly for high-grade or higher-staged tumors or those with positive surgical margins [2].

Systemic therapy is an area of active investigation for the treatment of ONB. Chemotherapy is usually administered as a combination of a platinum-based regimen combined with etoposide or another agent [2,53]. As discussed previously, many groups have advocated for the use of neoadjuvant chemotherapy, particularly for patients with orbital or intracranial extension as a means of decreasing the tumor burden prior to a planned definitive resection [11,41,42]. As adjuvant therapy alone, a recent SEER database study has suggested that chemotherapy does not provide an overall survival benefit [54]. However, many believe that chemotherapy may provide greater benefits in combination with radiation therapy, and further work is ongoing to evaluate the optimal role of chemotherapy in the treatment of ONB [2]. Lastly, immunotherapy is an emerging area of study for the treatment of ONB. A number of targeted therapies, including programmed death-ligand 1 (PD-L1) and transcription growth factor-beta (TGF-beta) blockade, somatostatin receptor 2 blockade, and peptide receptor radionuclide therapy are currently under investigation for the treatment of ONB [10,14,55].

## 8. Discussion

Olfactory neuroblastoma (ONB) represents a rare neuroendocrine malignancy arising within the sinonasal cavity from the olfactory neuroepithelium. ONB can be difficult to diagnose, as it frequently presents with a range of nonspecific sinonasal complaints, including nasal obstruction and epistaxis, and diagnosis can be accomplished through a combination of physical examination, nasal endoscopy with biopsy, and computed tomography and magnetic resonance imaging. Although it is historically approached via an open craniofacial approach, the current standard of care for primary tumor treatment is endoscopic resection with negative margins, with or without craniotomy, as necessary. Regional metastasis to the cervical lymph nodes may be detected at presentation or may occur in a delayed fashion and should be addressed through elective neck dissection or radiation. Adjuvant radiotherapy should be considered, particularly for high-grade tumors, advanced tumor stage, or positive surgical margins. Systemic therapy is an area of active investigation in both the neoadjuvant and adjuvant setting, with many advocating in favor of induction chemotherapy for significant orbital or intracranial involvement prior to surgical resection. Prolonged locoregional and distant surveillance are indicated following definitive treatment given the tendency for delayed recurrence and metastasis.

## 9. Pediatric ONB

Although primarily a disease affecting adults, pediatric patients present with ONB infrequently. As a result of the rarity of pediatric ONB, multi-institutional and database studies have been necessary to gain an understanding of this disease process. A 2020 Safi et al. systematic review encompassing 94 pediatric patients found a wide span of affecting ages (less than 1 year to 21 years old at diagnosis). Notably, Safi et al. reported that the most common Kadish stage at presentation was Kadish C (60.6%) compared to Kadish B (28.7%), Kadish D (8.5%), and Kadish A (2.1%). Neck disease was present in just over 20% of patients at diagnosis [56]. Similarly, a 2021 Berger et al. review of the National Cancer Database identified 45 pediatric patients with a mean age of 10.9 years. In contrast, Berger et al. identified only 48.9% of patients being Kadish C or D, with almost a quarter of patients being Kadish stage A at diagnosis, a distribution that approximated that of the adult population [5].

Just as in adults, the mainstay of therapy for pediatric ONB is surgical resection with negative margins, with consideration of adjuvant radiation and chemotherapy, as indicated. However, within the pediatric population, there is ongoing discussion regarding the utility of chemotherapy with Venkatramani et al., among others, proposing that pediatric ONB is responsive to chemotherapy [57]. Several small retrospective studies have reported the utilization of chemotherapy as a part of the treatment paradigm, either as neoadjuvant therapy prior to surgery or in combination with radiation as an adjuvant treatment, although there is not a clear consistently proposed regimen [57,58,59]. Berger et al. have reported a significantly higher rate of utilization of chemotherapy for pediatric patients (46.2%) than adult patients (18.8%). Overall, just as in adult patients, pediatric patients with ONB should be primarily managed with surgical resection with negative margins with consideration for adjuvant radiation or chemotherapy. However, the optimal role of chemotherapy has yet to be clearly elucidated and may play a larger role in years to come within the pediatric population.

## 10. Future Directions

There are a variety of exciting, ongoing areas of active research for ONB. Historically, advances in the care of patients with ONB have been limited largely by the rarity of the disease process. However, over recent years the combination of improved basic science techniques and multi-institutional collaborations have allowed for significant advances in the field. For example, large patient databases and emerging multi-institutional research consortia have been able to collate larger pools of patient data to provide greater insights regarding epidemiology of patients with ONB [19,20]. Based on this updated understanding, among other objectives, there are ongoing efforts to improve the existing ONB staging system to allow for more accurate and personalized prognostication [9].

From the perspective of surgical management, unilateral resection as an approach to preserve olfaction is an area of ongoing investigation. Single- and multi-institution retrospective reviews have reported encouraging results from both the perspective of oncologic outcomes, as well as olfactory preservation, but recent cadaveric work has suggested that patient selection will likely need to be further tailored as we gain a greater understanding of the connectivity of the olfactory system [43,44,45,46]. Overall, research focusing on unilateral resection and the preservation of olfaction in amenable patients represents an effort to tailor treatment to each individual patient and maximize quality of life while maintaining an oncologically sound surgical resection.

Additionally, there are numerous treatment avenues involving systemic therapy that are currently being investigated. For one, patients who present with extensive local disease involving the orbit have recently been considered for induction chemotherapy as a treatment approach aimed to preserve the orbit. Recent studies focusing on this treatment modality have been promising for orbital preservation, especially for higher Hyams-grade tumors that may respond better to systemic treatment [11,40,41,42]. Future work will better elucidate the role of induction chemotherapy compared to a tailored upfront surgical resection with adjuvant treatment as the best approach to maximize quality of life and oncologic outcomes for each individual patient.

Lastly, there are a host of different targeted immunotherapies that are being actively investigated for the treatment of recurrent or metastatic ONB. In the last few years, there have been efforts to identify novel targets for the treatment of ONB through gene expression profiling, such as by Romani et al. [55,60]. Such work has identified, in patients with aggressive disease, targetable molecular processes, such as angiogenesis pathways, the TGF-beta pathway, the IFN-alpha response, and the IL2-STAT5 and IL6-JAK-STAT3 signaling pathways. Separate works by Cracolici et al. and Lechner et al. have characterized somatostatin receptor 2 expression in up to 82.4% of ONB tumors, suggesting a role for somatostatin-based imaging and therapy [14].

Furthermore, modern techniques are being utilized to better understand ONB tumor heterogeneity at a molecular level. In particular, a 2018 Classe et al. integrative, multi-omics-based analysis of 59 ONB tumors identified two distinct tumor subgroups, including the *IDH2* R172 mutation-enriched basal-like subtype, as well as the neural-like subtype with genome-wide reprogramming and loss of DNA methylation at the enhancers of DNA axonal guidance genes [60]. Further work needs to be performed to elucidate the clinical and prognostic implications of these newly highlighted molecular markers, but there is well-founded hope that future care will entail a personalized approach based on tumor-specific markers for each patient.

Overall, there is a growing understanding of ONB transcriptomics and an emerging effort to target these pathways through a range of immunotherapies, including programmed death-ligand 1 (PD-L1) and transcription growth factor-beta (TGF-beta) blockade, somatostatin receptor 2 blockade, and peptide receptor radionuclide therapy [10,14,55].

All in all, these many areas of current investigation will hopefully provide greater insight into ONB patient epidemiology, molecular drivers of aggressive disease, the ability to preserve smell and vision without sacrificing oncologic outcomes, and targeted systemic therapy.

## Figures and Tables

**Table 1:** Modified Kadish staging system.

Stage A	Tumor confined to the nasal cavity
Stage B	Tumor confined to the nasal cavity and paranasal sinuses
Stage C	Tumor extent beyond nasal cavity and paranasal sinuses, including involvement of the cribriform plate, base of the skull, orbit, or intracranial cavity
Stage D	Tumor with metastasis to cervical lymph nodes or distant sites

**Table 2:** Dulguerov staging system.

T1	Tumor involving the nasal cavity and/or paranasal sinuses (excluding sphenoid), sparing the most superior ethmoidal cells
T2	Tumor involving the nasal cavity and/or paranasal sinuses (including the sphenoid) with extension to or erosion of the cribriform plate
T3	Tumor extending into the orbit or protruding into the anterior cranial fossa
T4	Tumor involving the brain

## Data Availability

No new data were created or analyzed in this study. Data sharing is not applicable to this article.
